# Six-Year Training Improves Everyday Memory in Healthy Older People. Randomized Controlled Trial

**DOI:** 10.3389/fnagi.2016.00135

**Published:** 2016-06-09

**Authors:** Carmen Requena, Agustín Turrero, Tomás Ortiz

**Affiliations:** ^1^Department of Psychology, Universidad de LeónLeón, Spain; ^2^Department of Biostatistics, Universidad Complutense de MadridMadrid, Spain; ^3^Department of Medical Psychology, Universidad Complutense de MadridMadrid, Spain

**Keywords:** older adult, everyday memory, long-term, training, randomized, trial

## Abstract

**Purpose of the study:** Everyday memory of older persons does not improve with intensive memory training programs. This study proposes a change in these programs based on a time-extended and massive intervention format.

**Design and Methods:** The sample of 1007 healthy older persons (mean age 71.85; SD = 5.12) was randomized into 2 groups. The experimental group followed an extended 6 years of training (192 sessions over 192 weeks) whereas the control group received an intensive training (3 sessions per week for a total of 32 sessions in 11 weeks). The program included cognitive and emotional content whose effects were assessed with the Rivermead Behavioral Memory Test (RBMT) and with the Mini-Mental State Examination (MMSE). Both groups were evaluated initially, after 32 sessions, and again after 6 years.

**Results:** The relative improvements measured with Blom’s derivative showed that everyday memory and mental status of the experimental group were significantly better both in the short (Δ% 8.31 in RBMT and Δ% 1.51 in MMSE) and in the long term (Δ% 12.54 in RBMT and Δ% 2.56 in MMSE). For everyday memory and mental level, the overall gain estimate representing the mean difference in pre-post change between time-extended and intensive groups was 0.27 (95% CI: 0.13–0.40) and 0.54 (95% CI: 0.40–0.67), respectively. Time-extended programs have significantly improved everyday memory in contrast with the usual intensive programs whose effects decay with time. There are also significant increases in mental level scores while daily life functionality is preserved in all subjects who completed the training.

**Implications:** These results suggest that it is possible to preserve everyday memory in the long term with continuous training and practice. Massive and time-extended formats may contribute in the future to a paradigm shift in memory programs for healthy older people.

## Introduction

The exponential growth of an aged population in the early 21st century means that not only has their overall life expectancy increased, but a far greater proportion are reaching this advanced life expectancy. Retirement thus occupies about one third of our whole lifetime and often coincides with the reduction of physical (Mullen et al., 2012) or cognitive activity (Bamidis et al., 2014), and/or a reduction in social activities (Wrosch et al., 2013). Therefore, aging societies face the challenge of preserving the autonomy of older people until the end of their lives. Since the brain and cognition remain plastic even in older age, this collective can improve their memory skills through instruction and practice (Mayr, 2008), even if some cognitive standards decline.

Early memory training approaches used mono-factorial techniques such as visualization or organization, cognitive re-structuring, concentration, faces and numbers, mnemonic techniques (Lachman et al., 1992), or the loci method (Rose and Yesavage, 1983). Ulterior mono-factorial approaches implement not only memory techniques but also train other related support processes such as attention, reasoning, and processing speed. In cognitive-training studies such as Advanced Cognitive Training for Independent and the Vital Elderly (ACTIVE), subjects are distributed into different groups, each of them training a particular process. The evaluation of each process as a laboratory task allows the measurement and comparison of the effect of both trained and non-trained processes. Yet, as evidence has accumulated regarding their benefits, interest in multifactorial approaches has increased since the efficacy of a given cognitive component may depend upon the activation and interaction of various processes (Gross et al., 2012).

Multifactorial programs are a jumble of several methods based on the observation that real-world tasks rarely depend on a single component of cognition. Accordingly, the cognitive-training approach was to train a range of cognitive processes, that are likely involved in many everyday tasks and that decline with age. For instance, the everyday activity of cooking requires a variety of cognitive processes including planning, attentional (executive) control, and working memory. Significant examples of multifactorial programs are centered on prospective memory training which is needed in daily life to remember errands and appointments or accurately remember medical information. These programs incorporate discussion groups which provide opportunities to overcome emotional alterations caused by erroneous beliefs about memory (Phillips and Ferguson, 2013). Additionally, the mutual support given by the group improves training performance (Wilson, 1992). Both mono-factorial and multifactorial programs are generally carried out in an intensive fashion, that is, 1–15 sessions given over 6–8 weeks.

Memory training programs have immediate beneficial effects over trained and distal processes and seem to be momentarily transferred to daily life activities. A recent meta-analysis on intensive memory-training programs shows that the tendency towards memory improvement does not seem to be associated with the specific trained content but rather with their diversity and repetition which also produces more solid effects on everyday life. The effects during the middle and long term of multifactorial programs is not known, hence the effect of their transfer and the persistence of their training benefits are also ignored. Concerning mono-factorial programs, their benefits are also immediate and since these improvements decay after 2 years, reinforcement sessions have been proposed as a means to maintain the longitudinal positive effect of mono-factorial programs. In particular, the ACTIVE program, a major randomized trial on cognitive training for older adults, shows gains in the training group as opposed to the control even 5 years after training. However, in the 10-year evaluation of the ACTIVE program, we found reinforcement sessions preserve certain improvements with respect to the basal line in some cognitive functions (reasoning and speed-of-processing), but not everyday memory which decays under the basal line. Therefore, the longitudinal follow-up of current intensive programs has made it evident that their benefits with regard to memory decline over time, principally because the majority of the participants do not continue to employ the techniques they have learned (Cohen-Mansfield, 2014).

The key to preserving everyday memory gains over time is the variety of content (Gross et al., 2012), the repetition of the training, and the number of sessions (Rebok et al., 2014). Our conceptual proposal is to set up a time-extended training program to train for both cognitive and emotional content, while simultaneously practicing them in real life. The objectives of this study were to contribute to the knowledge of the effect of time on mental level and everyday memory through the analysis of the effect of a time-extended training program vs. an intensive program as control.

## Materials and Methods

### Participants

**Figure 1:**
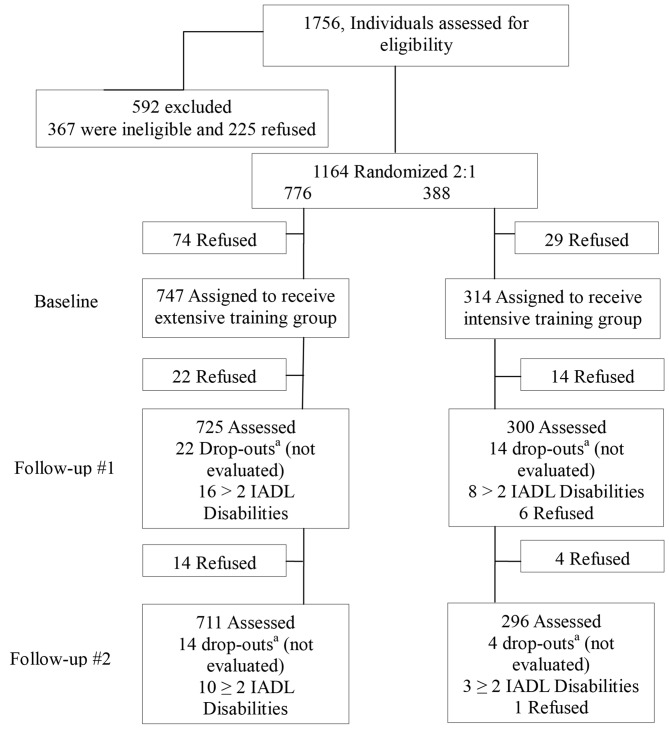
**Sample and flow.** ^a^Drop-outs between follow-up sessions.

The initial candidate group consisted of 1756 subjects older than 65 years. They were all living independently and enjoyed good functional and cognitive status. The participants were recruited through members of the city’s senior community centers for the retired established at the Ponferrada Town Hall, an urban district in the province of León, Spain. Of the total subjects interested in participating, 592 were excluded. Ninety-five percentage of the remaining participants completed the training intervention. Baseline characteristics are shown in Figure 1 according to intervention groups.

Finally, the study included 711 subjects in the experimental group and 296 in the control group (Figure 1). The demographic characteristics of the experimental group were: 617 women and 94 men whose ages ranged from 65 to 83 years old (average: 71.76, standard deviation (SD): 5.05); educational level: 95 had obtained a university degree, 136 had completed secondary school, and 480 had only finished primary school; marital status: 350 were married, 290 were widowed, 62 were single, and 9 were divorced. In the control group, the age range was from 65 to 83 years old (average: 71.85, SD: 5.12); 253 women and 43 men; educational level: 46 had obtained a university degree, 62 had completed secondary school, and 188 had only finished primary school; marital status: 173 of them were married, 90 were widowed, 27 were single, and 6 were divorced.

The exclusion criteria were: self-reported diagnoses of Alzheimer’s disease, severe sensory impairment (sight and/or hearing), moderate dependence (help needed to perform Instrumental Activities of Daily Living (IADLs) more than twice a day) reported by the social worker, or unavailability during the study period. Written, informed consent was obtained from all the participants after they received both verbal and written information about the study.

The trial was approved by the Ethical Hospital Service of León and Technical Committee of the City Council of Ponferrada. Subjects who did not meet the inclusion criteria were referred to a family doctor for further evaluation and check-ups.

### Procedure

#### Memory Training Program

The initial recruitment began in January 2006 with informative talks given in senior citizen community centers where the study was carried out. Those who signed up as prospective participants were later contacted by telephone and evaluation and appointments were scheduled. The evaluations lasted for approximately 90 min and involved the completion of a socio-demographic questionnaire and the administration of the tests chosen for this study. The initial response was greater than anticipated. Since we were limited by the capacity of the senior citizen community centers, this problem was overcome by publicly drawing lots of the interested subjects in order to decide who was to participate.

The subjects chosen were then randomized into two groups: extensive training (experimental) and intensive training (control). A randomized controlled procedure with a 2:1 allocation ratio was carried out combined with stratified randomization by age, sex and mini-mental state examination (MMSE) scores. It was decided that any subject who abandoned the study at any stage or who did not attend at least 80% of the sessions, would be excluded from the statistical analysis. This intervention has been registered^1^ and assigned the reference^2^. The control group received 32 intensive sessions, at a frequency of three times weekly for 11 consecutive weeks in 2006. The experimental group received 192 sessions, at a frequency of once weekly for 32 weeks from October to May each year between 2006 and 2012.

The program was carried out in two phases: the first phase analyzed the differences between the extended and intensive training programs after both groups had received 32 sessions; and the second phase analyzed the effect of the additional 160 sessions of training only received by the experimental group. In all, three assessments were performed: at baseline, after 32 training sessions (follow-up #1), and a final evaluation of both groups in year 6 (follow-up #2). In the case of the control group, follow-up #1 took place after 32 sessions at the 11th week, and follow-up #2 at the 6th year. In the case of the experimental group, follow-up #1 occurred after 32 sessions at the 32nd week, and follow-up #2 took place after the last (6th) year after receiving an additional 160 sessions in 160 weeks.

The training group in the time-extended program was comprised of a working group (which focused on common tasks) and a discussion group (which fostered active participation and experience exchange). Memory training program sessions were based on *the Group Memory Therapy Model* and on the *Memoria Mejor* (MM) program (Requena, 2002, 2005). The training program included the instruction of eight qualified psychologists (M.A. or Ph.D.). Therapists used register sheets for each participant on which correct/failed exercises were checked and registered relative to each training module: homework, group sessions, and attendance. This data was collated in therapists’ monitoring sessions at the end of each module. Further details on the administration, intervention, and monitoring of the memory training are offered^3^.

#### Group Memory Therapy

Wilson’s model includes cognitive and emotional content which we organized into 11 modules, the first nine of which addressed techniques and strategies to improve working memory both retrospective and prospective. During the last two modules, the affect of mood was addressed in the discussion groups.

##### Module I: How does the memory work?

The objectives of the memory program were explained as well as issues regarding the different types of memory and memory in older age. This module included home exercises so that participants could accurately measure their own performance in different memory tasks.

##### Module II: Making it easier to remember

External aids such as temporary storage (e.g., shopping list), long-term storage (e.g., address book), planning (e.g., calendar) or organizing one’s space (e.g., keeping each thing in its appropriate place) were explained.

##### Module III: Concentration

This module dealt with maintaining concentration skills such as having brief periods of rest during reading, suppression of external distractions (e.g., working in a calm room or doing one thing at a time), or working against one’s intrusive thoughts (e.g., verbalizing the action during its performance).

##### Module IV: Practice makes perfect

Information to be learned by the experimental subjects was presented in group settings (e.g., names of people or objects). Each group member identified information to learn and remember such as people’s names, objects, or dates. One of those elements was selected to practice daily using worksheets. This exercise was spaced throughout the day using a rule of doubling the time interval in between practice sessions (e.g., the exercises commenced at 10:30 am, 10:32 am, 10:36 am, 10:44 am, and so on: Wilson and Moffat, 1992).

##### Module V: Remembering to run errands

The group focused on exploring ways to reduce the chance of forgetting (e.g., method of Loci). The procedure consisted of creating an itinerary (almost always a sequence of rooms) that was very familiar to the subject. This itinerary was linked to tasks or issues that the experimental subject wished to remember (e.g., to do errands or make phone calls).

##### Module VI: Remembering information

Practicing this module entailed tasks such as recalling a newspaper article or recent news seen on television. Homework related to this was also given (e.g., fill-in-the-blank exercises on paper regarding the news).

##### Module VII: Active listening and expressing ideas

Cards with sequences of listening activities as well as instructions for expressing ideas were given to the participants. Each group member gave a presentation about a freely chosen topic. The cards were distributed among the group members to maintain a minimal rate of conversation and also help them to remember the presentation’s main issues.

##### Module VIII: Making the best use of my memory

Exercises within this module were designed to stimulate mental skills that reinforce memory. They included sensory stimulation exercises (e.g., improving visual acuity using a photograph), voluntary attention (e.g., identifying a misspelled word), intellectual structuring (e.g., re-ordering a disorganized text), language (e.g., word puzzles) or calculations (e.g., Sudoku).

##### Module IX: Exercising memory strategies

Training subjects were required to engage in categorization activities by grouping information. In order to remember a list of words, the subject had to organize them into different categories which required a degree of abstraction. During practice, a disorganized list of elements was given. Next, the participants had to sort the list into different categories. Finally, those words had to be remembered without naming the categories.

##### Module X: Confronting others’ problems

Many of the group members share similar worries including cognitive, emotional, family, economic, and legal problems. The group was encouraged to discuss the problems or struggles that were proposed by the psychologist or a group member. If any particular group member required specific information, he or she was referred to a social worker at the senior citizens community center.

##### Module XI: Emotion and memory

This module concentrated on the relationship between mood and memory performance (or more precisely the self-perception of memory performance). The therapy group discussed the relationship between confidence in one’s memory and factors like depression, good vs. bad days, and anxiety. Relaxation and auto-instructional training were proposed to aid memory when lapses occur.

### Pastimes in the MM Program

Pastimes in the MM Program were selected from journals and magazines by the older people themselves (Requena, 2002, 2005). Exercises improve linguistic, numeric, spatial and constructive tools. Pastimes are: (1) alphanumeric code; (2) extraction of words from other words; (3) word completion from missing vocals; (4) recognition of misplaced words; (5) alphabet soups; (6) peseta/euro conversion; (7) tangram (5 levels of difficulty); (8) domino; (9) magic stair; (10) crosswords combined with labyrinths; (11) knight moves; (12) operations with addresses; (13) calculation of prizes of fruit; (14) letter puzzles (1 level of difficulty); and (15) colors and forms layout patterns. Pastimes are also ordered in levels of difficulty with at least six individual exercises for each type of exercise. Exercise types with training, reinforcement, and solutions are available at the web address given below^4^. The contents of the 32 training sessions were organized in the following way: the first 11 training sessions corresponded with the 11 modules. Each of the first nine pastimes occupied a session (from the 12th to the 20th), while the remaining pastimes occupied two training sessions each (from the 21st to the 32nd). With regard to the 160 refresher sessions only received by the experimental group, they were distributed in 32 sessions during the following 5 years after the treatment (1 weekly session from October to May). The contents of refresher had different individual exercises but were organized in the same manner as the training sessions.

The temporal distribution of training sessions was as follows: sessions were held over 75 min in groups of between 8 and 10 people, 60% of this time was set aside for modules and pastimes, 30% of the session involved debate and discussion concerning the difficulty of the exercises and its daily life application, while the remaining 10% of the session was dedicated to solving doubts raised by homework exercises which were repetitions of already trained abilities.

### Measures

A number of instruments was used to evaluate the psychological effects of the extended training. The Mini-Mental Cognitive Examination (MEC-35) test is the Spanish adaptation (Lobo et al., 1979; Lobo, 1987) of the MMSE (35 items; Folstein et al., 1975). This test is widely used to quantify intellectual deterioration or mental level and its progression over time since it can be used repeatedly and thus document an individual’s response to training or treatment. Mental level is measured with tasks involving orientation, attention, concentration, language, calculation, constructive praxia, and work memory. A measure equal or higher than 1.5 times the SD with respect to the subject’s normative levels (age and education), implies a sucessful mental level. The MMSE has an 84.6% sensitivity and an 82% specificity (Saz and Lobo, 1993).

Memory was evaluated through the standardized measure Rivermead Behavioral Memory Test (RBMT; Wilson et al., 1985). The RBMT is a battery designed to tap the participant’s memory doing everyday tasks. There is evidence that favors the use of the RBMT in older adults and for neuropsychological assessment of memory impairment (Cockburn, 1996). The RBMT assesses different types of memory such as associative memory, prospective memory, visual memory, verbal memory, topographic memory, control, and recognition strategies which produces a global score from 0 to 12 points. A Spanish version of the RBMT has been used and validated with the Wechsler Memory Scale.

### Statistical Analysis

The scores for each cognitive or functional measure were normalized using the Blom transformation (Blom, 1958; Lehmann, 1975), the most commonly used rank-based inverse normal transformation. Homogeneity for the experimental and control groups at baseline was analyzed using two-sample *t*-tests for transformed measures and age, and using χ^2^ (chi squared) tests to assess sex and educational level. In order to evaluate the effects of the memory program, a repeated-measures-mixed-effects model was used, with the group as the between-subjects factor (experimental and control) and the repeated measures were the MMSE and RBMT. Mental status and everyday memory were measured at baseline, post-training (follow-up #1), and at the follow-up evaluation (follow-up #2). A Bonferroni *post hoc* analysis was completed. All statistical analyses were carried out using SPSS 22 statistics software.

#### Effect Size Calculation

Effect size was defined as “gain” in order to adapt to standard usage in the relevant literature (Gross et al., 2012; McDaniel et al., 2014; Rebok et al., 2014). Training gain was calculated in three stretches, follow-up #1 − baseline, follow-up #2 − baseline, and follow-up #2 − follow-up #1. Average differences were divided by the pooled SD to place gain values of all memory training programs in the same scale. The same calculations were performed to obtain control group gains.

Retest-adjusted gains were also calculated as experimental improvement from baseline to year 6 minus control improvement from baseline to year 6 divided by the intra-subject SD of the composite score. The first set of effect sizes were standardized differences in mental level and everyday memory change between baseline and follow up #1 and follow up #2 assessments. In contrast, retest-adjusted effect sizes or gains represent everyday memory and mental level change attributable to training by adjusting for a retest effect in control.

#### Improvement

A first assessment of the long-term change in mental level (as measured by MMSE) and everyday memory is given by the relative percentage increase in these measures at the two time points evaluated: follow-up #1 and follow-up #2. This measure is defined by: Δ% measure follow-up-up # 1 equal to intermediate measure minus baseline measure divided by baseline measure and multiplied by 100. The same calculation to follow-up #2 was repeated.

The values of these increases for the MMSE and RBMT scores along the period of study are shown in Table 1. The columns represent the average increase/decrease of each measure in relation to the baseline scores for each group. For example, from the baseline values the MMSE scores increased in the experimental group an average of 1.51% and 2.56% in follow-up sessions #1 and #2 respectively. By contrast, in the control group this average value remained virtually unchanged during follow-up session #1 (0.06%) and had decreased slightly in follow-up session #2 (−0.20%). This divergent tendency was also evident for the RBMT measures.

**Table 1:** **Mean values, standard deviations (SDs) and mean relative percentage increases for mini-mental state examination (MMSE) and rivermead behavioral memory test (RBMT) at baseline and follow-up sessions**.

		MMSE	SD	RBMT	SD
	Baseline	29.35	0.98	7.26	2.26
Training group	Follow-up #1	29.78	0.94	7.70	2.27
(*n* = 711)	Follow-up #2	30.09	0.89	7.98	2.31
	Baseline	29.32	1.09	7.22	2.26
Control group	Follow-up #1	29.32	0.94	6.97	2.29
(*n* = 296)	Follow-up #2	29.25	1	7.04	2.15
**Mean relative**			**Δ%**		**Δ%**	
**percentage increases**			**MMSE**		**RBMT**	
	Follow-up #1		1.51%		8.31%	
Training group	Follow-up #2		2.56%		12.54%	
	Follow-up #1		0.06%		−2.42%	
Control group	Follow-up #2		−0.20%		−0.87%	

*MMSE, Mini-Mental State Examination; RBMT, Rivermead Behavioral Memory Test; SD, Standard deviation*.

#### Effects of the Memory Training Program

**Figure 2:**
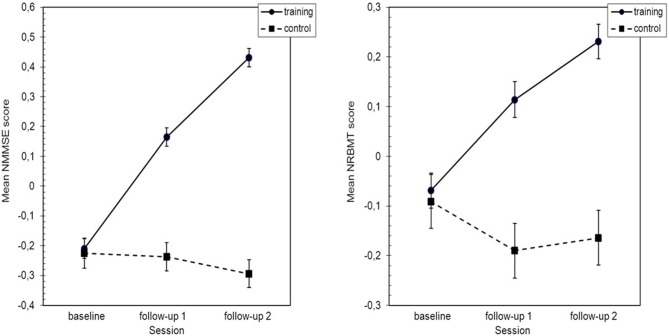
**Standarized means plot of normal blom composite of the mini-mental state examination (NMMSE) and normal blom composite of the rivermead behavioral memory test (NRMBT) scores.** Error bars indicate SEM. NMMSE, Normal Blom composite of the Mini-Mental State Examination; NRBMT, Normal Blom composite of the Rivermead Behavioral Memory Test; SEM, Standard Error of mean.

To evaluate the effects of this intervention program, a repeated-measures model was used. When the plot of the means for Normal Blom composite of the Mini-Mental State Examination (NMMSE) was analyzed (Figure 2), it appeared that the experimental and control groups exhibited divergent behavior during the follow-up period.

There is also a clear NMMSE * group interaction. Statistical analysis highlighted the significant differences among NMMSE scores through the follow-up period (*F*_(2,1946)_ = 92.12, *p* < 0.001 η^2^ = 0.125). The interaction of the NMMSE * group was very significant (*F*_(2,1946)_ = 139.31, *p* < 0.001 η^2^ = 0.194) and there were significant differences between the NMMSE scores in both groups (*F*_(1,1005)_ = 53.28, *p* < 0.001 η^2^ = 0.05).

When the means of Normal Blom composite of the Rivermead; Behavioral Memory Test (NRMBT) scores were plotted (Figure 2), it again seemed that the experimental and control groups exhibited divergent behavior during the follow-up period and that an NRBMT * group interaction was present. The statistical analysis showed significant differences among NRBMT scores during the follow-up period (*F*_(2,1951)_ = 24.26, *p* < 0.001 η^2^ = 0.045) and a strongly significant NRBMT * group interaction (*F*_(2,1951)_ = 69.76, *p* < 0.001 η^2^ = 0.107). There were also significant differences between the NRBMT scores in both groups (*F*_(1,1005)_ = 14.97, *p* < 0.001 η^2^ = 0.015; see Figure 2).

Means and SDs of transformed measures and age at baseline are shown in Table 2. NMMSE and NRBMT represent the transformed values of these measures. None of the comparisons between the means of the two groups reflected statistically significant differences when subjected to a *t*-test (all *p* > 0.73). Also, the groups were homogeneous with regard to gender (*χ*^2^_1_ = 03, *p* = 0.58) and educational level (*χ*^2^_2_ = 1.563, *p* = 0.458).

**Table 2:** **Multiple comparisons for normal blom composite of the mini-mental state examination (NMMSE) and normal blom composite of the rivermead behavioral memory test (NRBMT)**.

		NMMSE			NRBMT		
	Session	Mean	SD	95% CI	Mean	SE	95% CI
Training	Baseline	−0.210	0.033	(−0.274, −0.147)	−0.069	0.035	(−0.138, −0.000)
group	Follow-up #1	0.164	0.031	(0.104, 0.224)	0.114	0.036	(0.044, 0.184)
	Follow-up #2	0.431	0.031	(0.371, 0.491)	0.231	0.035	(0.162, 0.300)
Control	Baseline	−0.225	0.05	(−0.324, −0.126)	−0.091	0.054	(−0.198, 0.015)
group	Follow-up #1	−0.237	0.047	(−0.331, −0.144)	−0.190	0.055	(−0.298, −0.082)
	Follow-up #2	−0.294	0.047	(−0.387, −0.201)	−0.164	0.055	(−0.271, −0.057)

	**Session**	**Gains**	**SD**	**95% CI**	**Gains**	**SE**	**95% CI**

Training	Follow-up #1−baseline	0.421	0.051	(0.320, 0.522)	0.186	0.051	(0.086, 0.286)
group	Follow-up #2−baseline	0.721	0.053	(0.618, 0.824)	0.309	0.051	(0.208, 0.409)
	Follow-up #2−Follow-up #1	0.310	0.051	(0.210, 0.411)	0.119	0.051	(0.019, 0.219)
Control group	Follow-up #1−baseline	−0.014	0.082	(−0.175, 0.147)	−0.106	0.082	(−0.267, 0.056)
	Follow-up #2-baseline	−0.083	0.082	(−0.244, 0.079)	−0.078	0.082	(−0239, 0.082)
		−0.070	0.082	(−0232, 0.091)	0.027	0.082	(−0.134, 0.186)

Retest adjusted gain	Follow-up #2−follow-up #1	0.537	0.069	(0.401, 0.673)	0.269	0.069	(0.134, 0.219)

*Means, standard errors (SE), effect size (gain), retest effect and 95% confidence intervals (CI). NMMSE: Normal Blom composite of the Mini-Mental State Examination. NRBMT: Normal Blom composite of the Rivermead Behavioral Memory Test. Gain: Effect size defined as the mean difference between the post-training and pre-training scores for the trained group divided by the pooled standard deviation (SD). Positive effect sizes indicate improvement. Retest-adjusted gain was defined as training improvement from baseline to year 6 minus control improvement from baseline to year six divided by the pooled standard deviation. CI, Confidence Interval*.

The *post hoc* analysis indicated that there were significant differences between the means of the NMMSE scores for both groups in the follow-up #1 and follow-up #2 sessions. There were also significant differences among the means of the baseline, intermediate, and final NMMSE scores in the experimental group. No significant differences were found among the means of NMMSE scores in the control group. All of these comparisons were made using the Bonferroni test at the 5% level and the results of these comparisons are shown in Table 2.

The *post hoc* analysis emphasised the significant differences between the means of NRBMT scores in both groups at the follow-up #1 and follow-up #2 sessions. There were significant differences between the means of NRBMT values at baseline and in the 2nd follow-up sessions but not between the follow-up #1 and follow-up #2 sessions in the experimental group. No significant differences were found among the means of NRBMT scores in the control group. All comparisons were made with the Bonferroni test at the 5% level of significance, as shown in Table 1.

Longitudinal gains with respect to the three measures of time and retest-adjusted gain, are also shown in Table 2. The interpretation of data follows Cohen’s values, which describe an effect size of 0.2 as small, 0.5 as medium, and 0.8 as large (Brown and Prescott, 2006). Because the analyses included three comparisons, a corrected significance level of *p* < 0.05 was used.

#### Descriptive Analysis of Cognitive Subdomains in the Follow-Up Period

**Figure 3:**
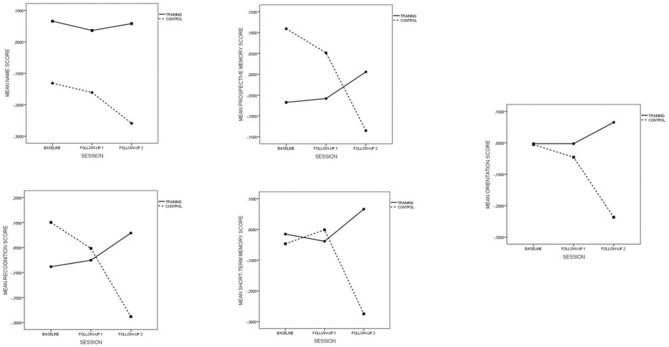
**RBMTs Subdomains in the follow-up period**.

Global RBMT measures include 12 items distributed in five subdomains: “names” (items 1 + 2), “prospective memory” (items 3 + 4), “recognition” (items 5 + 7), “short-term memory” (items 6 + 8 + 10), and “orientation” (items 9 + 11 + 12). Statistical analyses of standardized means plots are shown in Figure 3.

In view of the plots, control and training groups differ significantly in the baseline scores in the subdomains “names,” “prospective memory,” and “recognition” (*p* < 0.001), therefore results concerning these subdomains should be interpreted with caution. The subdomain “names” exhibits a divergent behavior between the two groups in the follow-up #2, but the differences shown in the baseline do not allow one to interpret the differences observed.

Regarding the subdomains “prospective memory” and “recognition” which had higher baseline scores in the control group, 2 facts are worth mentioning: the divergent trend in both groups and the strongly significant differences at follow-up session #2 (*p* = 0.009 and *p* < 0.0001 respectively) were again noted.

Finally, the “short-term memory” and “orientation” subdomains are homogeneous in the baseline (*p* = 0.597 and *p* = 0.961 respectively) with significant differences found in follow-up session #2 (*p* < 0.001 in both cases) but not in the follow-up session #1 (*p* = 0.525 and *p* = 0.48 respectively).

Plots and *p*-values displayed come from the repeated measures analyses for each subdomain, once such measures were Blom-transformed. The analysis of subdomains is therefore consistent with the global RBMT score, that is, with the long-term gradual improvement.

**Figure 4:**
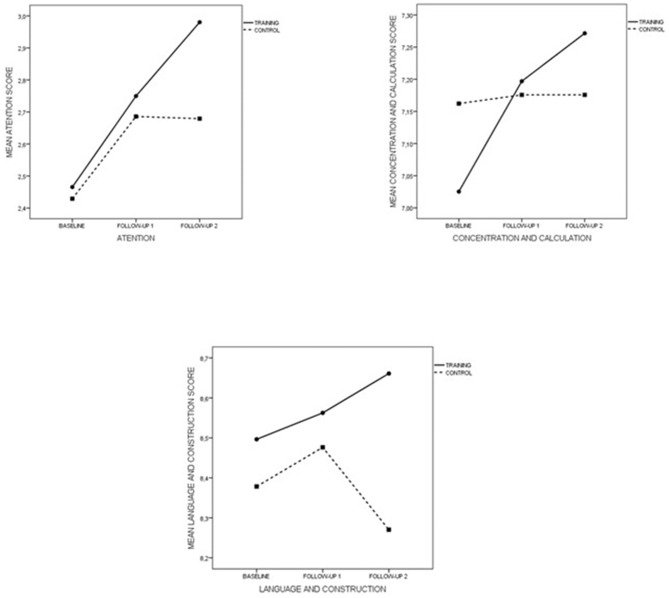
**MMSEs Subdomains in the follow-up period**.

The MEC-35 test contains five subdomains: orientation, short-term memory, attention, concentration and calculation, and language and construction. The first two subdomains have already been analyzed with RBMT items, while the three final ones have been statistically analyzed; their standardized means plot is shown in Figure 4.

Control and training groups differ significantly in the baseline scores in the subdomain “concentration and calculation” (*p* = 0.018), whereas the other two were homogeneous in baseline (*p* = 0.29 and *p* = 0.175). For these two subdomains, the evolution along the follow-up period was similar for both groups up to follow-up #1. From here on, the behavior was divergent with significant differences in follow-up #2 (*p* < 0.001 in both cases). Results relative to “concentration and calculation” clearly showed the stabilization of scores along the follow-up period in the control group and the significant growth of these scores along the follow-up period in the training group (*p* < 0.001 in both times). In addition, differences between groups shown at follow-up #2 for this subdomain were close to the significance (*p* = 0.058).

## Discussion

As far as we know, this is the first study to analyze the effects of a time-extended memory training program which included cognitive and emotional content for older adults. The extended format resulted in significant short and long-term improvement in everyday memory and mental level (as measured by MMSE), in contrast with the usual intensive format whose effect on memory decays with time. All participants have preserved independent performance in IADLs with respect to the basal line.

The behavior of the variables “mental level,” MMSE, and “everyday memory,” RBMT, at follow-up are remarkably divergent between the experimental (extended program) and the control groups (intensive program; see Figure 2). The descriptive analysis of that behavior shows that the average percentage increase in MMSE scores in the experimental group reaches 1.51% and 2.56% after 32 weeks and 6 years respectively. On the other hand, in the control group the above-mentioned average remains invariant and decreases slightly (0.20%) at both periods (see Table 2). The average relative percentage increase in RBMT scores in the experimental group was 8.31% and 12.54% after 32 weeks and 6 years respectively, while in the control group this percentage decreased on average 2.42% and 0.87% at both periods (see Table 2).

In the first phase of our study, both experimental and control groups were given the same total number of sessions and the same content but with different formats: extended and intensive, respectively. The extended form of the training resulted in a significant gain in both mental level (0.421) and everyday memory (0.186) in relation to their corresponding baseline scores (follow-up #1−baseline), against the invariance or non-significant decrease of both scores with the intensive training (mental level: −0.014, everyday memory: −0.106). In the second phase of the study, only subjects in the experimental group received further sessions in an extended format. Now, MMSE scores exhibit gains of 0.721 with respect to those obtained at the baseline (follow-up #2−baseline) while RBMT scores show gains of 0.309 in relation to the base line (follow-up #2−baseline). Again the intensive training or control group did not show any gains in both measures: −0.083 (follow-up #2−baseline).

These results can first be compared with short term training, since immediate effects of cognitive multifactorial programs are well known in the literature. For example, in the meta-analysis on memory programs for healthy older adults, the overall gain of memory was 0.31 (Gross et al., 2012), and 0.14 in the research on the combined effects of cognitive and erobic memory training (McDaniel et al., 2014). The only longitudinal program with which to compare results on long-term memory is the mono-factorial trial ACTIVE, whose immediate memory improvement is well established, but which decays after 6 months (Neely and Bäkman, 1993) or after 2 years (Ball et al., 2002). Intensive programs with booster sessions such as ACTIVE preserve memory levels after five years (performance gains of 0.23), but not longer (Willis et al., 2006; Goh et al., 2012). The recent 10 years follow-up study with ACTIVE shows improvement in reasoning and processing speed, but benefits are dispelled in everyday memory which decays under the basal line with gains of 0.06 (Rebok et al., 2014). In contrast, the overall gain of our time-extended training after 6 years was 0.54 for mental level and 0.27 for everyday memory, which are manifestly higher than any intensive program (see Table 2). As ACTIVE researchers acknowledge, it is possible that more extensive practice or greater dosing are required to reach durability in memory performance (Rebok et al., 2014).

The long term decay of memory in ACTIVE shows that either a multifactorial program, or more reinforcement sessions, or more extended duration of the training, or all three conditions together are required to reach durability in memory performance as verified by the results of this study. Probably, the long term preservation of memory requires not only the repetition of reinforcement sessions, but also a multifactorial and durable program. Moreover, preserving memory in the long term depends not only on variables internal to the memory program, but also on the posterior practice of trained abilities in real life (Bennett et al., 2014). Contrasting with most intensive programs, the time-extended program includes a module devoted to home tasks promoting the continued practice of trained content in real-world situations.

Significant post-training differences measured at follow-up #1 after receiving the same number of sessions leads us to conclude that the temporal format of the program determines the training effect on healthy older people. However, the extended format itself is an insufficient condition for the long-term success of memory programs since leisure activities and IADLs also have this format but do not systematically improve the cognitive measures of older people. This is the case of formative leisure activities which happen to be associated with the preservation of everyday memory while non-formative leisure activities like card games do not sustain the preservation of memory. (Requena and López, 2014). Similarly, the retrospective and prospective memory training is associated with benefits to IADLs such as cooking, while verbal memory practice is not.

Therefore, the continuous improvement in mental level and everyday memory during the program is not only explained by the temporal format, but also by the explicitly multifactorial nature of the training. This approach is based on the observation that both cognitive and real-world daily functions rarely depend on a unique cognition component. For example, the daily activity of cooking requires a variety of cognitive processes including planning, attention, work memory, and prospective memory (Craik and Bialystok, 2006). In this regard, a key component of our cognitive training was to train a wide variety of cognitive abilities which are involved in many daily tasks and which decay with age. This multifactorial nature of the program explains why the same divergent tendency is observed in the experimental group as with the control group which has already been observed on global MMSE and RBMT scores (see Figures 3, 4). Contrastingly, ACTIVE was designed to analyze the benefits of mono-factorial programs over specifically trained cognitive abilities. Viewed in this way, it is not surprising that improvements in reasoning and processing speed led to improved performance measures of memory and IADLs after training and reinforcement sessions. These results are consistent with the thesis that multiple cognitive abilities are more likely to have an effect on IADL performance.

Special consideration should be given to the high compliance of the experimental group with the intervention program, since 95% of participants completed the training. In contrast, the ACTIVE retention rate was 44% among subjects who were booster-trained and 20% among subjects who were non-booster-trained. The low withdrawal rate of the experimental group may be due to either the intrinsic opportunity for social interaction or having the obligation of “something to do” (Morack et al., 2013). These aspects are reflected in our sample characteristics: lack of activity, availability, and scarcity of other opportunities to exercise cognitive and social functions (Zinke et al., 2014). On the other hand, special attention has been paid to strengthening the attendance and dealing with ruminations and false beliefs about memory in discussion groups in the extended program.

In summary, the results at 6 years demonstrate that a time-extended program with emotional and cognitive content has beneficial effects on everyday memory, mental level, and IADL function. The research has some limitations due to its multifactorial character since there is an inherent difficulty in attributing particular improvements to specific properties of the program. Future research on time-extended memory programs will determine how to adjust the measures and training into everyday functional tasks. Another concern is the practical sustainability of the intervention in terms of its costs.

## Author Contributions

CR has conceived and defined the investigation; involvement includes all research phases: design methodology, sample collection, monitoring, implementation of the program in memory training and preparation of the article. AT has claimed responsibility for the statistical analysis of research. TO has collaborated in the methodology and writing of the article.

## Funding

This research has been partly founded by the Ayuntamiento de Ponferrada (Spain).

## Conflict of Interest Statement

The authors declare that the research was conducted in the absence of any commercial or financial relationships that could be construed as a potential conflict of interest.

## Footnotes

^1^www.controlled-trials.com

^2^http://www.isrctn.com/ISRCTN46109513

^3^http://www.isrctn.com/ISRCTN46109513

^4^http://envejecimientoentodaslasedades.unileon.es/primera-generacion.html
